# Trends in suicide mortality rates in the Republic of Cyprus between 2004 and 2020: changes in age, gender and suicide method

**DOI:** 10.1192/bjo.2024.770

**Published:** 2024-10-10

**Authors:** Andreas Chatzittofis, Nicos Middleton, Maria Karanikola

**Affiliations:** Medical School, University of Cyprus, Nicosia, Cyprus; and Department of Clinical Sciences/Psychiatry, Umeå University, Umeå, Sweden; Department of Nursing, School of Health Sciences, Cyprus University of Technology, Limassol, Cyprus

**Keywords:** Suicide mortality rates, suicide, Cyprus, suicide method, violent suicide method

## Abstract

**Background:**

The Republic of Cyprus has recorded the greatest increase in suicide mortality among Eastern Mediterranean countries, with an average annual increase of 5.1% in 2000–2019.

**Aims:**

To investigate trends in suicide mortality rates between 2004 and 2020 in the Republic of Cyprus, with a focus on age, gender and suicide methods.

**Method:**

Suicide deaths (ICD-10 taxonomy, including ‘undetermined’ code) and population denominators were obtained from the National Mortality Registry and Statistical Office, respectively. Directly standardised (European Standard) mortality rates were calculated for four gender and age groups. Annual change was estimated using Poisson regression models with interaction terms to assess differential trends over different time periods.

**Results:**

There were 560 suicide deaths; these were four times more frequent in men, and approximately 80% were classified as ‘violent’ for both genders. The male suicide rate doubled from 4–5 to 9–10 per 100 000, mostly before 2012, representing a 9% annual change (rate ratio = 1.09, 95% CI 1.03, 1.15; *P* = 0.002). From 2013, the trend reversed (effect modification *P* < 0.001) with a 4% annual decrease (95% CI −9%, 1%). Declines were not uniform across all age groups; rates in males aged 45–64 years continued to rise, surpassing the previously high rate in males aged 25–44 years. Rates in females declined from 4–5 per 100 000 to 2–3 over the study period. Overall, the male-to-female suicide rate ratio was 5.33 (95% CI 3.46, 8.19) in 2017–2020, compared with 2.73 (1.88, 3.95) in 2004–2008.

**Conclusion:**

Although suicide rates remain relatively low, the gender differential has widened in the Republic of Cyprus. Further analysis of trends in relation to unemployment and other socioeconomic indicators is warranted.

Suicide mortality rates across the world vary significantly and are influenced by numerous factors, at both the individual and population levels.^1,2^ Population-level factors include socioeconomic and sociopolitical variables, alongside factors at the healthcare system level factors, such as access to mental healthcare services and effectiveness of suicide prevention policies.^3,5^

Several studies have focused on age, gender and suicide methods.^1,6^ A study investigating suicide rate trends in years 2000–2019 estimated the global age-standardised suicide rate (ASR) in 2019 to be 9.0 per 100 000 population for both genders, whereas this was 12.6 in males and 5.4 in females.^2^ The ASR per 100 000 population in Europe in 2019 for both genders was 10.5; the highest ASR was reported in Lithuania (20.2) and the lowest in the Republic of Cyprus (3.2), Greece (3.6), Albania (3.7) and Italy (4.3).^2^ Nevertheless, the Republic of Cyprus and Greece were among those with the highest average annual percent changes in suicide rate, with increases of 5.1 and 2.6%, respectively.^2^ Further investigation into age and gender trends and factors associated with this rise is warranted to understand the changing patterns and to design and implement targeted suicide prevention strategies.^2^

Consistently and universally, higher suicide rates are recorded in males compared with females.^2,7^ During the past 20 years, a decrease has been reported in suicide rates for both genders in most European countries; yet in Greece, The Netherlands and Portugal, a statistically significant increase in ASR was reported for both genders. A significant increase in ASR was reported for females in Norway and males in the Republic of Cyprus.^2^ Similarly, different suicide mortality trends have been reported across age groups globally and across Europe.^1^ Suicide rates in 1990–2015 remained stable or declined slightly in those aged 15–64 years and decreased significantly in those aged 65+ years in the Czech Republic, Belgium, France, Italy, Spain and Germany, whereas they increased in all age groups in Greece.^1^

Suicide method, mainly categorised as violent and non-violent, is an important factor to be considered in suicide prevention strategies.^6,9^ Violent methods aim to cause immediate death and include self-shooting, hanging, jumping from a height, self-poisoning with pesticides or drowning. Non-violent methods, such as drug overdose or self-cutting, are less likely to cause immediate death.^8^ Stable patterns in suicide methods may allow for targeted preventive strategies. However, temporal changes in suicide methods pose challenges and may reflect variations in access to specific suicide means and a need to revise preventive measures accordingly.^7^

Besides the annual report of the World Health Organization, to the best to our knowledge, there has been only one (outdated) study in the international literature reporting on suicide trends in the Republic of Cyprus, referring to the period 1988–1999.^10^ This study reported very low suicide rates, with increasing tendencies in both genders. Lack of studies originating from the Republic of Cyprus may be partly attributed to mortality monitoring systems. Although the Cyprus Health Monitoring Unit (Ministry of Health of the Republic of Cyprus) has recorded mortality rates since 2004, including mortality due to suicide, no study regarding suicide trends in the Republic of Cyprus has been published to date. The period from 2004 to the present includes the global financial crisis (2008) and the national financial crisis (2012–2013), both associated with impaired mental health and increased suicide rates.^11,13^

Understanding epidemiological trends and patterns in suicide is essential for developing effective interventions and preventive strategies that should be tailored to the specific needs of each country and population.^7^ The aim of this study was to examine trends and patterns in suicide mortality rates in the Republic of Cyprus between 2004 and 2020 with a focus on age, gender and suicide methods.

## Method

Ethics approval was not required for this study.

Following ICD-10 taxonomy, suicide deaths data were obtained from the Mortality Registry, and population denominators were recorded from the National Statistical Office of the Republic of Cyprus. Events coded as of ‘undetermined intent’ were also included in the analysis; however, these were very low in number. Indeed, the number of events classified as undetermined has consistently been very low in the Republic of Cyprus; only 50 such events were reported in the 17-year period 2004–2020, with no indication of change in the use of these codes during this period. Although the numbers are low to allow any inference, this appears consistent with the conclusion of Rocket et al that the Republic of Cyprus is not among the countries in which the ratio of undetermined intent mortality to suicide is indicative of a high potential for undercounting through the use of this code.^14^

Directly standardised mortality rates were calculated for four gender/age groups using the European Standard population. Counts and frequencies of suicide deaths were also calculated according to gender, age group, mode of death classification (X70–X83 ICD-10 codes for ‘violent’; X70–X83 ICD-10 codes for ‘non-violent’; X84 ICD-10 codes for ‘unspecific means’), district of residence and season across (a) the whole period (2004–2020) and (b) over four time periods: 2004–2008, 2009–2012, 2013–2016 and 2017–2020. Owing to statistical imprecision in the estimates as a result of small numbers, smooth 3-year moving averages are presented in the plots.

Annual change was estimated using Poisson regression models with interaction terms to assess differential trends over time periods. Rate ratios with 95% confidence intervals were calculated to represent annual change over time periods, e.g. 2004–2012, 2013–2020. The extent to which the estimated trend differed between comparison periods was assessed by including a ‘year × period’ interaction term in the models (*P*-value for effect modification).

## Results

There were 560 recorded suicides; suicides were four times more frequent in men (*n* = 452, 80.7%) compared with women (*n* = 108, 19.3%). The male/female ratio of suicides ranged from 73.1% in 2004–2008 to 83.4% in more recent years. The majority of male suicides (72.3%) occurred in the age group of 24–64 years. There were 69 suicides in males aged 15–24 years (15.3%) and 56 suicides in males aged over 65 years (12.4%). Nearly four in five (79.6%) suicides in women were in the age group of 24–64 years, with 12 (11.1%) and ten suicides (9.3%) pertaining to the age groups of 15–24 years and over 65 years, respectively. Table 1 presents counts and frequencies of suicides according to gender, age-group, mode of death classification, district of residence and season across (a) the whole study period and (b) over four time periods.

**Table 1:** Counts of suicide deaths (*N* = 560) according to gender, age, mode of death classification (violent versus non-violent), district of residence and season over four time periods in 2004–2020

	2004–2020^a^	2004–2008	2009–2012	2013–2016	2017–2020	*P*-value
Gender						
Male	452 (80.7%)	76 (73.1%)	117 (83.6%)	133 (80.6%)	126 (83.4%)	
Female	108 (19.3%)	28 (26.9%)	23 (16.4%)	32 (19.4%)	25 (16.6%)	0.15
Age group by gender						
Males (two missing information)						
15–24 years	69 (15.3%)	10 (13.2%)	18 (15.5%)	20 (15.0%)	21 (16.8%)	
24–44 years	174 (38.7%)	34 (44.7%)	48 (41.4%)	50 (37.6%)	42 (33.6%)	
45–64 years	151 (33.6%)	24 (31.6%)	35 (30.2%)	43 (32.3%)	49 (39.2%)	
Over 65 years	56 (12.4%)	8 (10.5%)	15 (12.9%)	20 (15.0%)	13 (10.4%)	0.80
Females						
15–24 years	12 (11.1%)	1 (3.6%)	4 (17.4%)	4 (12.5%)	3 (12.0%)	
25–44 years	43 (39.8%)	12 (42.9%)	8 (34.8%)	13 (40.6%)	10 (40.0%)	
45–64 years	43 (39.8%)	13 (46.4%)	9 (39.1%)	12 (37.5%)	9 (36.0%)	
Over 65 years	10 (9.3%)	2 (7.1%)	2 (8.7%)	3 (9.4%)	3 (12.0%)	0.95
Mode of death classification by gender						
Males						
Violent	405 (89.6%)	60 (79.9%)	100 (85.5%)	125 (94.0%)	120 (95.2%)	
Non-violent	47 (10.4%)	16 (21.1%)	17 (14.5%)	8 (6.0%)	6 (4.8%)	<0.001
Females						
Violent	86 (76.6%)	20 (71.4%)	18 (78.3%)	28 (87.5%)	20 (80.0%)	
Non-violent	22 (20.4%)	8 (21.7%)	5 (21.7%)	4 (12.5%)	5 (20.0%)	0.49
Method of suicide by gender						
Males						
Hanging	183 (40.5%)	23 (30.3%)	44 (37.6%)	54 (40.6%)	62 (49.2%)	
Drowning	3 (0.7%)	0 (0.0%)	0 (0.0%)	1 (0.8%)	2 (1.6%)	
Firearms/explosives	111 (24.6%)	19 (25.0%)	30 (25.6%)	36 (27.1%)	26 (20.6%)	
Jumping from height	67 (14.8%)	9 (11.8%)	17 (14.5%)	24 (18.1%)	17 (13.5%)	
Pesticides	32 (7.31%)	12 (15.8%)	11 (9.4%)	5 (3.8%)	4 (3.2%)	
Other poisons	19 (4.2%)	4 (5.3%)	9 (7.7%)	4 (3.0%)	2 (1.6%)	
Other method	37 (8.2%)	9 (11.8%)	6 (5.1%)	9 (6.8%)	13 (10.3%)	0.01
Females						
Hanging	33 (30.6%)	7 (25.0%)	5 (21.7%)	12 (37.5%)	9 (36.0%)	
Drowning	5 (4.6%)	1 (3.6%)	2 (8.7%)	1 (3.1%)	1 (4.0%)	
Firearms/explosives	0 (0.0%)	0 (0.0%)	0 (0.0%)	0 (0.0%)	0 (0.0%)	
Jumping from height	39 (36.1%)	9 (32.1%)	9 (39.1%)	14 (43.8%)	7 (28.0%)	
Pesticides	9 (8.3%)	6 (21.4%)	2 (8.7%)	1 (3.1%)	0 (0.0%)	
Other poisons	13 (12.0%)	2 (7.1%)	3 (13.0%)	3 (9.4%)	5 (20.0%)	
Other method	9 (8.3%)	3 (10.7%)	2 (8.7%)	1 (3.1%)	3 (12.0%)	0.35
District of permanent residence^b^						
Nicosia	172 (34.3%)	25 (28.7%)	41 (34.5%)	57 (37.0%)	49 (34.8%)	
Limassol	144 (28.7%)	34 (39.1%)	31 (26.1%)	42 (27.3%)	37 (26.2%)	
Larnaca	85 (17.0%)	16 (18.4%)	23 (19.3%)	25 (16.2%)	21 (14.9%)	
Paphos	69 (13.8%)	11 (12.6%)	16 (13.5%)	18 (11.7%)	24 (17.0%)	
Famagusta	31 (6.2%)	1 (1.2%)	8 (6.7%)	12 (7.8%)	10 (7.1%)	0.44
Non-permanent	32 (6.0%)	0 (0.0%)	14 (10.5%)	11 (6.7%)	7 (4.7%)	0.07
Season – both genders						
Winter	114 (20.4%)	28 (26.9%)	25 (17.9%)	30 (18.2%)	31 (20.5%)	
Spring	149 (26.6%)	20 (19.2%)	48 (34.3%)	39 (23.6%)	42 (27.8%)	
Summer	161 (28.8%)	27 (26.0%)	30 (21.4%)	60 (36.4%)	44 (29.1%)	
Autumn	136 (24.3%)	29 (27.9%)	37 (26.4%)	36 (21.8%)	34 (22.5%)	0.06
Season – males						
Winter	98 (21.7%)	23 (30.3%)	24 (20.5%)	27 (20.3%)	24 (19.1%)	
Spring	121 (26.8%)	12 (15.8%)	39 (33.3%)	31 (23.3%)	39 (31.0%)	
Summer	128 (28.3%)	18 (23.7%)	25 (21.4%)	50 (37.6%)	35 (27.8%)	
Autumn	105 (23.2%)	23 (30.3%)	29 (24.8%)	25 (18.8%)	28 (22.2%)	0.02
Season – females						
Winter	16 (14.8%)	5 (17.9%)	1 (4.4%)	3 (9.4%)	7 (28.0%)	
Spring	28 (25.9%)	8 (28.6%)	9 (39.1%)	8 (25.0%)	3 (12.0%)	
Summer	33 (30.6%)	9 (32.1%)	5 (21.7%)	10 (31.3%)	9 (36.0%)	
Autumn	31 (28.7%)	6 (21.4%)	8 (34.8%)	11 (34.4%)	6 (24.0%)	0.26

a. Not including an additional 32 and 18 deaths in men and women respectively coded as ‘of undetermined intent’.

b. Not including 37 events with unknown or incomplete information. Furthermore, 32 were classified as non-permanent residents and hence were included as a separate category. According to the 2021 census, the distribution of population by district is as follows: Nicosia 38%, Limassol 28%, Larnaca 17%, Paphos 11% and Famagusta 6%. In 2011, the equivalent figures were: 39%, 28%, 17%, 10% and 5%, respectively.

The proportion of suicide deaths classified as violent was high in both males (89.6%) and females (76.6%). A gradual increase was observed in males from 79.9% in 2004–2008 to 95.2% in 2017–2020 (*P* < 0.001). Most of this increase could be attributed to an increase in hanging, which accounted for 49.2% of all suicides in 2017–2020, after gradual rises from 30.3% in earlier years (*P* = 0.01). No clear inferences can be drawn in the case of females owing to the smaller number of events. However, hanging was the most common suicide method in both females (one in three) and males (one in two) in the years 2017–2020. By contrast, use of pesticides decreased in 2017–2020 in both genders.

Regarding geographical distribution, proportions were largely consistent with the distribution of the population denominators across the five districts of the Republic of Cyprus. Although there were increases in suicides in Nicosia (the capital city and largest district) and Paphos (the smallest district), the differences were not statistically significant (*P* = 0.44).

Α seasonality pattern was evident in males, with more suicides occurring in the spring and summer months. Although the pattern did not appear to be systematic across all study periods, this seasonality has become more evident over the years in men (*P* = 0.02). No clear seasonality pattern was observed in females; this could be attributed to the smaller number of suicides, which may not permit inferences.

**Fig. 1:**
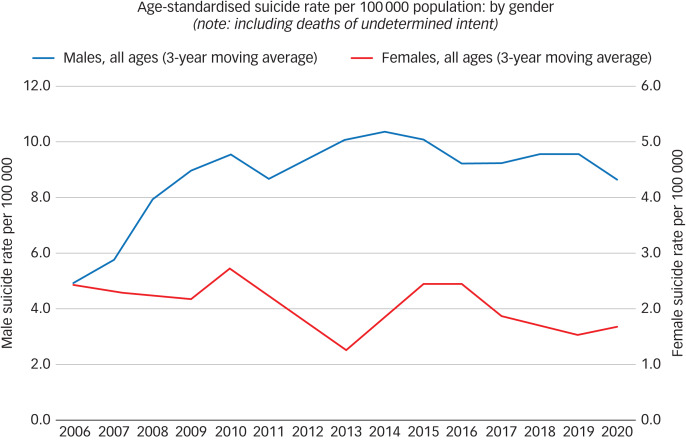
Age-standardised suicide rate per 100 000 population by gender, 2004–2020 (3-year moving averages).

Figure 1 presents trends in gender-specific ASR per 100 000 population over the period 2004–2020. These rates incorporate events ‘of undetermined intent’; however, excluding them did not alter the findings (results not shown in detail). Overall, male ASR doubled over the study period: from 4–5 suicides per 100 000 in 2004, there was a sharp increase in all male age groups to 9–10 deaths per 100 000 by 2012–2013. Over the 2004–2012 period, the observed annual increase in male rates was 9% (rate ratio = 1.09 per year, 95% CI: 1.03, 1.15; *P* = 0.002). Male rates showed a 4% decrease (95% CI: −9%, 1%) from 2013 onwards. Although the decreasing trend over this period was not statistically significant (*P* = 0.114), there was clear evidence of a differential trend between 2004–2012 and 2013–2020 (effect modification *P* < 0.001), suggesting that the previously increasing suicide rate did not continue to rise. By contrast, the overall (all ages) female ASR showed a declining trajectory. The trend was not uniform over the whole period but was steeper or more gradual at different time points. Overall, female ASR decreased from 4–5 deaths per 100 000 in the earlier years to 2–3 in the later years. The observed annual decrease in female rates was 6–7% in both the earlier (2004–2012) and the later (2013–2020) periods, with no evidence of a differential trend (effect modification *P* = 0.90). Table 2 presents rate ratio estimates (95% CI) representing annual change in each gender/age group's suicide rates as calculated by Poisson regression models over 2004–2012 and 2013–2020, based on the overall temporal patterns.

**Table 2:** Trends in gender- and age-specific suicide mortality: rate ratios for annual change in periods 2004–2012 and 2013–2020

	Annual change, 2004–2012	Annual change 2013–2020	*P*-value for effect modification
	Rate ratio per year (95% CI)	Rate ratio per year (95% CI)	
Gender			
Male	1.09 (1.03, 1.15)*	0.96 (0.91, 1.01)	<0.001
Female	0.94 (0.85, 1.03)	0.93 (0.83, 1.04)	0.90
Age group by gender			
Males			
15–24 years	1.15 (0.99, 1.32)	0.99 (0.86, 1.13)	0.126
25–44 years	1.10 (1.02, 1.09)**	0.93 (0.85, 1.02)	0.006
45–64 years	1.05 (0.95, 1.15)	1.02 (0.93, 1.11)	0.67
Over 65 years	1.08 (0.91, 1.27)	0.85 (0.73, 0.99)**	0.039
Females			
15–24 years	1.09 (0.79, 1.50)	0.89 (0.64, 1.24)	0.38
25–44 years	0.92 (0.79, 1.07)	0.93 (0.77, 1.11)	0.95
45–64 years	0.93 (0.81, 1.07)	0.95 (0.79, 1.13)	0.88
Over 65 years	0.91 (0.68, 1.21)	0.94 (0.61, 1.53)	0.89

* *P* < 0.01.

**P < 0.05.

Over the whole period, male rates were four times higher than female rates (rate ratio = 4.10, 95% CI: 3.36, 4.99; *P* < 0.001). Increase in male rates with parallel decrease in female rates resulted in widening gender differential over this 17-year period. The M/F ratio was 5.33 (95% CI: 3.46, 8.19) in 2017–2020, compared to 2.73 (95% CI: 1.88, 3.95) back in 2004–2008 (Table 3).

**Table 3:** Male-to-female ratio and age-distribution of suicide mortality rates over four time periods during 2004–2020

	2004–2020	2004–2008	2009–2012	2013–2016	2017–2020	*P*-value for effect modification
	Rate ratio (95% CI)	Rate ratio (95% CI)	Rate ratio (95% CI)	Rate ratio (95% CI)	Rate ratio (95% CI)	
Gender						
Male	4.10 (3.36, 4.99)*	2.73 (1.88, 3.95)*	4.75 (3.13, 7.21)*	4.25 (2.91, 6.18)*	5.33 (3.46, 8.19)*	0.094
Female	1.00 (ref)	1.00 (ref)	1.00 (ref)	1.00 (ref)	1.00 (ref)	
Age group by gender						
Males						
15–24 years	0.77 (0.59, 1.01)	0.54 (0.29, 0.98)**	0.68 (0.40, 1.16)	0.81 (0.48, 1.35)	1.21 (0.72, 2.04)	0.49
25–44 years	1.00 (ref)	1.00 (ref)	1.00 (ref)	1.00 (ref)	1.00 (ref)	
45–64 years	1.06 (0.86, 1.31)	0.89 (0.56, 1.39)	0.89 (0.35, 1.17)	1.03 (0.69, 1.55)	1.50 (0.99, 2.27)***	
Over 65 years	0.66 (0.49, 0.90)	0.51 (0.25, 1.05)***	0.64 (0.35, 1.17)	0.84 (0.50, 1.40)	0.63 (0.34, 1.17)	
Females						
15–24 years	0.60 (0.33, 1.11)	0.26 (0.06. 1.13)***	0.93 (0.39, 3.02)	0.71 (0.23, 2.18)	0.73 (0.48, 2.90)	0.96
25–44 years	1.00 (ref)	1.00 (ref)	1.00 (ref)	1.00 (ref)	1.00 (ref)	
45–64 years	1.41 (0.95, 2.09)***	1.50 (0.76, 2.98)	1.57 (0.65, 3.80)	1.36 (0.64, 2.90)	1.18 (0.47, 2.90)	
Over 65 years	0.57 (0.31, 1.06)***	0.59 (0.20, 1.80)	0.77 (0.21, 2.84)	0.46 (0.13, 1.61)	0.55 (0.15, 1.99)	

* *P* < 0.001.

** *P* < 0.05.

*** *P* < 0.1.

**Fig. 2:**
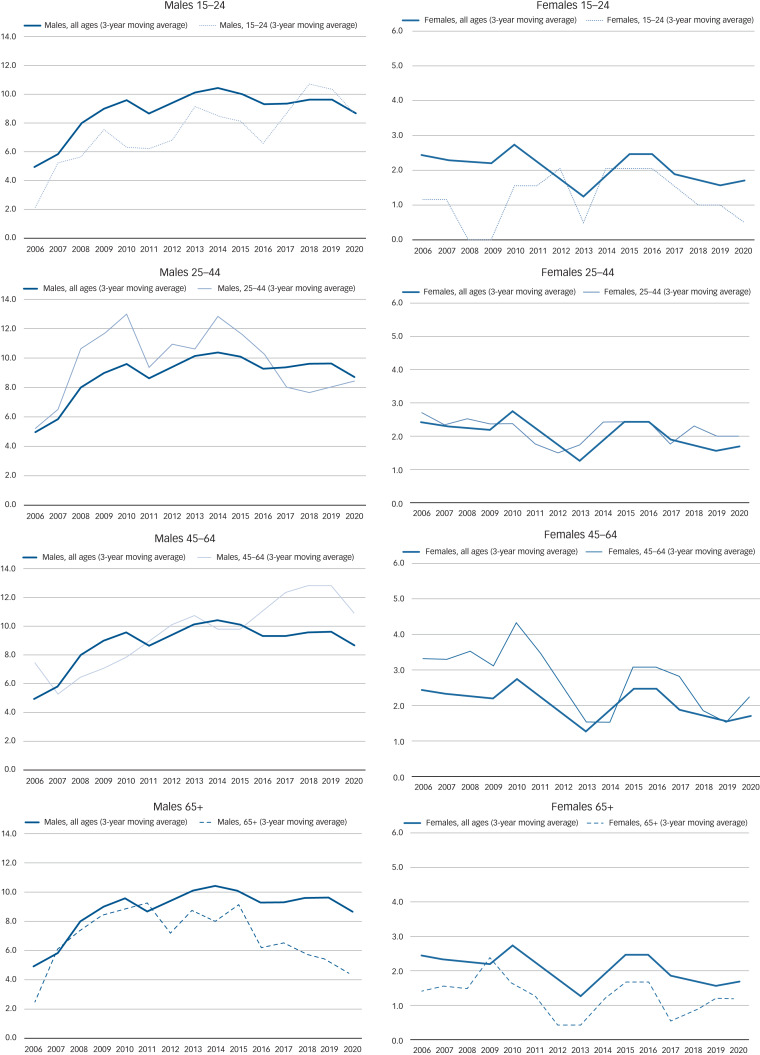
Trends in age-standardised suicide rate per 100 000 population by gender and age-group, 2004–2020 (3-year moving averages).

Figure 2 presents trends in ASR per 100 000 in 2004–2020 by gender and age group (15–24, 25–44, 45–64 and 65+ years); relevant data are also presented in Table 2. To facilitate comparisons, the overall suicide rate was superimposed on all figures.

A rise in ASR in 2004–2012 was recorded in all four age groups in males. Suicide rates in males aged 25–44 years showed a steep and statistically significant 10% increase per year in this period (rate ratio = 1.10, 95% CI: 1.02, 1.09; *P* = 0.019). However, ASR showed an annual decline of 7% in 2013–2020, reaching a plateau in recent years (rate ratio = 0.93, 95% CI: 0.85, 1.02; *P* = 0.12). The differential trend in 2004–2012 (increase) compared with 2013–2020 (decrease) was statistically significant (effect modification *P* = 0.006). A smaller but steady annual increase of around 2–5% in ASR in males aged 45–64 years was recorded over the whole study period, rising from 6–8/100 000 population in the early years to 12–13/100 000 population in recent years. Owing to this steady rise, together with the observed reversal of the trend in males aged 25–44 years, suicide rates were higher among males aged 45–64 years in the most recent time period than in males 25–44 years, which was the age group with the highest rates for most of the earlier study period.

The steepest rise among males in relative terms was recorded among the younger age group, with a 15% annual increase (95% CI: −1%, 32%); this increasing trend over the first period was only marginally statistically significant (*P* = 0.057). No clear pattern was observed in the second half of the study period (2013–2020). Suicide rates also showed an upward trajectory among males aged over 65 years in the first period, whereas there appeared to be a steady and statistically significant decreasing trend of 15% in suicide rates per year after 2013 (rate ratio = 0.85 95% CI: 0.73, 0.99). Owing to this statistically significant decrease in the older age group, males aged over 65 years had the lowest rate in recent years (4/100 000) compared with all other age groups.

The temporal patterns were more mixed in females. With the exception of the younger age group, which showed a non-statistically significant increase in 2004–2012, all other age groups had decreases in rates up to 2012, even if these were preceded by small increases in some cases up to 2008–2009. Following 2013, the year with the lowest rates across all age groups, female rates appeared to rise, but the trend seemed to reverse after only a few years. Over the long term, there was a decreasing trend in female suicide rates. Although this trend was not statistically significant, most likely owing to the small number of events, there was no evidence of a differential trend between the earlier and latest period in any of the age groups.

Given the decreasing trend from 2013 onwards in rates among men aged 25–44 and over 65 years, in parallel with rises in rates among men aged 15–24 and 45–64 years, the age distribution of suicide appears to have changed over the study period for men. Specifically, in the early years of the study period, men aged 25–44 years had the highest rate of all age groups. Rates in men aged 45–64 years were only slightly lower, but they were twofold lower among both men aged 15–24 years (rate ratio = 0.54 95% CI: 0.29, 0.98; *P* = 0.043) and those over 65 years old (rate ratio = 0.51 95% CI 0.25, 1.05; *P* = 0.067), relative to men aged 25–44. Rates among the 45–64 year age group have exceeded those of the 24–44 age-group by 1.5 times (rate ratio = 1.50 95% CI: 0.99, 2.27; *P* = 0.053) in recent years. Furthermore, owing to continuing increases, by 2017–2020, young men aged 15–24 years were the group with the second highest suicide rate; this was 1.21 times higher (95% CI: 0.72, 2.04) than the rate recorded among men aged 25–44 years in 2017–2020, although the observed difference was not statistically significant (*P* = 0.48). By contrast, the age distribution does not seem to have changed in females. Over the whole period, the age group with the highest rates among women was 45–64, followed by 25–44 years. However, the age differential in women appeared to be larger in the earlier period compared with the later period, suggesting a convergence of female suicide rates to around 1–2 per 100 000 across all age groups by the latest period (Table 3).

## Discussion

To the best of our knowledge, this is the first study to investigate trends in suicide mortality rates in the 17-year period from 2004 to 2020 in the Republic of Cyprus. There were four times more suicides reported in men, and approximately eight out of ten suicides were classified as violent in both genders.

The male suicide rate doubled in the study period, with most of the increase in 2004–2012. The trend reversed in 2013–2020, although the declines were not uniform across all age groups. Rates in males aged 45–64 years continued to rise in 2013–2020, surpassing the previously high rate in males aged 25–44 years. One explanation for this may be that those aged 45–64 years were more severely affected by the financial crises of 2008 and 2012 compared with those aged 25–44 years; younger adults may have had fewer family and economic responsibilities and been less vulnerable to the financial crisis. Similarly, a study looking at suicide across 58 countries after the 2008 financial crisis reported that the highest increase in suicide rates was in younger European men aged 15–24 years and in American men aged 45–64 years.^14^ Furthermore, a contributing factor to the observed rise in male suicide rates might be the increase in substance-use-related disorders among men during the past two decades in the Republic of Cyprus.^15,16^ Indeed, there was an escalation in the use of illegal substances in the study period, especially until 2010. Also, no gender-specific suicide prevention strategy was implemented to counteract the impact of the financial crises, or of other relevant factors in the Republic of Cyprus.

Moreover, the proportion of violent suicides in males showed a gradual increase from 2004 to 2016. The most common suicide method in men was hanging, followed by firearms and jumping, indicating a shift in suicide method in the decades compared. The most frequent suicide methods in men during 1988–1999 were self-poisoning, self-shooting and hanging, whereas those in women were jumping from height, self-poisoning and hanging.^10^ The rising rate of male suicide by hanging in 2014–2016 could be attributed to preventive efforts focused on limiting access to firearms and pesticides, as reported in Japan and Brazil.^17,18^ Suicide by hanging is also less likely to be misclassified as unintentional or undetermined intent, providing more accurate estimations. However, the number of events classified as undetermined was very low in the Republic of Cyprus. Overall, hanging replaced non-pesticide poisoning as the most common suicide method in the majority of 58 countries investigated between 2000 and 2015.^7^ Nevertheless, access to the means and cultural context are important determinants of the choice of suicide method; thus, there is a need to tailor preventive strategies accordingly.^17^ For instance, targeted interventions regarding access to army firearms were effective in preventing suicide by army firearms in Switzerland.^19^ The fact that self-shooting was the second most frequent suicide method in males in the present study, similar to findings in Brazil,^18^ could be attributed to the widespread availability of firearms owing to the hunting culture and the militarisation of the Republic of Cyprus. Importantly, men in the Republic of Cyprus are issued firearms, as they remain conscripted in the National Guard until the age of 50 years. However, no women were reported to have used a firearm as a method of suicide during the study period, although they also have extensive access to firearms; nearly every household has military and or hunting firearms. Thus, cultural and/or biological notions may contribute to women using less violent suicide methods.

A seasonality pattern with more suicides in spring and summer months in males was found herein, confirming previous studies in countries of the northern hemisphere.^20,21^ Proposed explanations include the seasonality of psychiatric disorders, which is related to bioclimatic factors such as sunlight, and augmented vulnerability over certain periods due to increased social and outdoor activity.^22^

The present declining trajectory in suicide rates in females is in contrast to the previously reported increasing trend in both genders between the years 1988 and 1999.^10^ It is unclear whether this decline can be attributed to improved mental health status in the female population and/or supportive and mental healthcare services.^15,23^ The highest suicide rate was in females aged 45–64 years, followed by those aged 25–44 years. However, it is difficult to draw conclusions owing to the small number of events. Whereas there was no statistical evidence of a differential trend between the earlier and latest period, there seems to have been a change over the past 35 years in the age profile of suicides in females; previous data in the Republic of Cyprus (1988–1999) reported that the highest suicide rate in females was in the 15–24 age group.^10^

Although future studies should also assess rates of non-suicidal self-harm, the present findings suggest a changing profile in terms of suicide methods among females, with violent methods becoming more prevalent. On the other hand, the declining trajectory in female suicide rates presented herein widened the male-to-female suicide rate ratio, although this remains relatively low compared with those of other European countries.^2^ Although gender differences in suicide rates vary among countries, it is well established that men have higher suicide rates than women, and this is mainly attributed to the use of methods with higher lethality, which allow for limited life-supporting interventions.^13^ Women most often commit drug overdose, with increased survival, which may reflect their decreasing suicide mortality rates over the years, worldwide.^24,25^ However, this cannot be supported herein, as the most frequent suicide method in women was jumping from height, followed by hanging; these are both violent methods. In addition, cultural norms, comorbidities and other sociopolitical variables may differently affect suicidal behaviour in females compared with males.^2,7^ Alternative explanations include the impact of the economic crises of 2008 and 2012 in terms of unemployment and financial difficulties, with more severe effects on men's mental health compared with that of women.^13^

The present suicide rate in both genders in the Republic of Cyprus is among the lowest in Europe and Eastern Mediterranean countries.^2^ The extent to which this may be attributed to underreporting is unclear. However, legislation in the Republic of Cyprus demands that police investigate all violent or unnatural deaths and those with unknown cause, which may decrease underreporting. Specifically, the police in the Republic of Cyprus are entitled to investigate the context and the circumstances related to a death of equivocal intent, yet this does not include a structured psychological autopsy, as is implemented in other countries. In addition, suicides by drug overdose are difficult to detect, as strong corroborative evidence, such as a suicide note and positive psychiatric history, is typically lacking. Other factors related to underreporting may include the social, cultural and religious context of the Republic of Cyprus. Specifically, the majority of population is registered as Christian Orthodox.^26^ However, Christianity may be also related to underreporting owing to social stigma and adverse funeral-related implications following suicide. Although there was no official change in the attitude of the Greek-Cypriot Orthodox Church to suicide during the period under study, anecdotal data report a more open attitude. Overall, further research is needed in countries that are unrepresented in the suicide literature.

Strengths of this study include the national coverage, using rigorous statistics from the Health Monitoring Unit of the Ministry of Health of the Republic of Cyprus, which is the official provider of Mortality Registry data. Despite the long timeframe of 17 years, which permitted investigation of temporal trends and patterns, the small numbers of suicides, especially in females, made interpretation of the results difficult; this was mainly because some results did not reach the level of statistical significance owing to lack of power. Furthermore, although official data statistics were used, the possibility of underreporting of suicides cannot be excluded. However, the mortality data statistics used herein are of the highest possible rigour (Ministry of Health and police). In addition, the incorporation of ‘undetermined intent’, with no changes in the results, can be considered a strength of the study.

Nevertheless, concealed suicides are an important issue; these include cases of undetermined intent, ill-defined causes of death and accidental deaths, mainly due to drowning and poisoning.^27^ The range of these cases varies significantly across countries. The only available relative data regarding the Republic of Cyprus are from 2006, in the context of an international comparative study.^14^ Of course, the magnitude of undercounting of suicide rates is relevant not only in the context of international comparisons but also in that of exploring temporal patterns in a national context, especially if there are changes in recording practices and sociocultural attitudes towards suicide stigma. While acknowledging that the present study provides the first report of suicide trends in the Republic of Cyprus in recent years, it is important to highlight that future studies should also consider any temporal patterns in accidental and ill-defined deaths in order to shed more light on these issues. Most importantly, age, gender and cause-specific death statistics in the Republic of Cyprus are available only upon request from the national Health Monitoring Unit; this is in contrast to many European countries, where there is open access to aggregate mortality statistics by cause of death.

According to the only available official statistics, it seems that there has been a steady decline in the percentage of ill-defined deaths relative to all deaths, from 9.1% in 2002–2006 to 3.7% in 2018–2020 (https://www.moh.gov.cy/). Although this decrease may be indicative of improved quality of recording, no inference can be drawn regarding the extent to which a higher or a lower degree of undercounting of suicides was taking place in earlier periods. At the same time, the present findings suggest that the increase in suicides occurred mostly before 2012, when the ratio of ill-defined to all deaths was still high, and this ratio continued to decrease in parallel with recorded declines in suicides in the most recent years. Finally, in terms of accidental deaths, the latest report in the Republic of Cyprus, in 2022, showed that deaths recorded as accidental drowning accounted for 9.1% (*n* = 30) of all causes of death, with half of these deaths reported in tourists, and deaths recorded as accidental poisoning accounted for 4.2% (*n* = 14) of all deaths. Although these data were not reported by official sources, it seems that the ratios of accidental deaths by drowning and poisoning to deaths by all causes range between 6.4–11.7% and 4.1–5.8%, respectively, without any clear increasing or decreasing pattern.

In conclusion, although the gender differential in suicide mortality in the Republic of Cyprus is relatively low compared with that of other European countries, it has widened, with steep rises in all male age groups up to 2012, a continuing rise in males aged 45–64 years and declines in female rates over the same period. Nevertheless, periodic fluctuations in suicide rates have been reported internationally. For example, data from the United States of America indicate that suicide rates increased by 37% between 2000 and 2018 then decreased by 5% between 2018 and 2020, before nearly returning to their peak in 2021. Several factors, including economic conditions, societal changes and public health interventions, can influence these fluctuations. In addition, data on comorbidity, especially in terms of physical illnesses highly correlated with suicide, were not included in the present study. Thus, further research should include the aforementioned factors in analysing suicide trends over time to reveal underlying causes and develop effective preventive strategies. Further analysis of trends in relation to unemployment and other socioeconomic indicators is warranted, as well as continuous monitoring of trends, as the study period did not include the COVID-19 pandemic owing to delays in suicide data recording. Although monitoring trends and patterns of suicide is deemed to be a duty of those employed in the Ministry of Health, suicide mortality is not reported systematically in the Republic of Cyprus, and the only published study in the literature is more than two decades old. The present study has the potential to stimulate further research and discussion on suicide prevention efforts in the Republic of Cyprus and in countries with similar contexts. Identifying the temporal trends and changing profile of suicide in the Republic of Cyprus highlights the need for a national suicide prevention strategy. Efforts should concentrate on the implementation of specific measures targeting risk groups according to available resources and considering the specific legal and cultural context. Continuous monitoring of suicide trends and patterns will also enable the evaluation of suicide prevention measures.

## Data Availability

The data that support the findings of this study are available from the corresponding author, A.C., upon reasonable request.
